# L-Carnitine and Mildronate Demonstrate Divergent Protective Effects on Mitochondrial DNA Quality Control and Inflammation Following Traumatic Brain Injury

**DOI:** 10.3390/ijms26072902

**Published:** 2025-03-22

**Authors:** Artem P. Gureev, Veronika V. Nesterova, Polina I. Babenkova, Mikhail E. Ivanov, Egor Y. Plotnikov, Denis N. Silachev

**Affiliations:** 1 Department of Genetics, Cytology and Bioengineering, Voronezh State University, 394018 Voronezh, Russia; gureev@bio.vsu.ru (A.P.G.); n3sterova.vero@yandex.ru (V.V.N.); ms.babenkova@bk.ru (P.I.B.); 2 A.N. Belozersky Institute of Physico-Chemical Biology, Lomonosov Moscow State University, 119992 Moscow, Russia; neokarda@mail.ru (M.E.I.); plotnikov@belozersky.msu.ru (E.Y.P.)

**Keywords:** traumatic brain injury, mitochondrial DNA, L-carnitine, mildronate, inflammation, gut microbiome, gene expression

## Abstract

Traumatic brain injuries (TBIs) are a serious problem affecting individuals of all ages. Mitochondrial dysfunctions represent a significant form of secondary injury and may serve as a promising target for therapeutic intervention. Our research demonstrated that craniotomy, which precedes the experimental induction of trauma in mice, can cause considerable damage to mitochondrial DNA (mtDNA), disrupt the regulatory expression of angiogenesis, and increase inflammation. However, the reduction in the mtDNA copy number and glial activation occur only after a direct impact to the brain. We explored two potential therapeutic agents: the dietary supplement L-carnitine—a potential reserve source of ATP for the brain—and the cardiac drug mildronate, which inhibits L-carnitine but activates alternative compensatory pathways for the brain to adapt to metabolic disturbances. We found that L-carnitine injections could protect against mtDNA depletion by promoting mitochondrial biogenesis. However, they also appeared to aggravate inflammatory responses, likely due to changes in the composition of the gut microbiome. On the other hand, mildronate enhanced the expression of genes related to angiogenesis while also reducing local and systemic inflammation. Therefore, both compounds, despite their opposing metabolic effects, have the potential to be used in the treatment of secondary injuries caused by TBI.

## 1. Introduction

Neurodegenerative diseases are a broad group of dysfunctions affecting the nervous system. They constitute the second leading cause of death and the primary cause of severe long-term disability worldwide. With rare exceptions, most of these diseases predominantly affect the elderly and are rarely encountered in younger individuals [1]. However, TBIs are an exception to this rule. TBIs can affect both infants and working-age adults. It is estimated that between 64 and 79 million people worldwide suffer from TBIs of varying severity each year [2].

The pathogenesis of TBI is characterized by a combination of primary and secondary injuries that lead to persistent neurological deficits. The primary stage of injury is caused directly by mechanical impact, resulting in diffuse axonal injury, the formation of hematomas, the loss of the integrity of the blood–brain barrier (BBB), and impaired blood flow [3]. The secondary phase can last from several days to even months and includes various types of damage, including mitochondrial impairment. Neuronal depolarization, which begins in the first hours after TBI, stimulates the release of excitatory neurotransmitters, leading to an increased intracellular calcium concentration, which can mediate various mitochondrial dysfunctions [4]. The disruption of the balance between pro-oxidant and antioxidant mitochondrial systems causes significant oxidative stress, resulting in damage to various mitochondrial and cellular components. This, in turn, exacerbates secondary injuries and can contribute to the development of other neurodegenerative diseases, including stroke [5] and Alzheimer’s disease [6].

One of the most sensitive components of mitochondria is mtDNA. This molecule is located in the mitochondrial matrix, in close proximity to the source of reactive oxygen species (ROS) in the mitochondria, and is not protected by histones. It also has a limited number of repair systems compared to nuclear DNA [7]. Currently, there is no clear understanding of the changes in the structural integrity of mtDNA in the brain that occur following TBI. The number of breaks in the mtDNA chain has not been investigated, and only a limited number of studies have focused on the analysis of mtDNA deletions. Research by Lifshitz and McIntosh (2003) showed that no increase in mtDNA deletions was detected after TBI. The authors suggested that this might be related to the protocol for isolating mitochondria, which could have eliminated mitochondria carrying mtDNA damage [8]. Another study on postmortem human brain samples also did not reveal an increase in mtDNA deletions after TBI [9], but this type of analysis is associated with certain challenges due to the degradation of mtDNA, which begins as early as 4–6 min after death [10].

The main therapeutic strategies focus on reducing the severity of secondary injuries following TBI, including the correction of mitochondrial dysfunctions. The development of new strategies to adapt neuronal mitochondrial metabolism to the altered conditions resulting from brain damage is an important challenge. L-carnitine, which is currently a popular dietary supplement, facilitates the transport of fatty acids across mitochondrial membranes for subsequent oxidation. Numerous reports indicate the neuroprotective effects of exogenous L-carnitine in various neurological and psychiatric disorders [11]. L-carnitine may reduce brain edema after TBI [12], prevent BBB disruption under methamphetamine-induced toxicity [13], provide acetyl-CoA as a substrate for brain energy metabolism, and also increase the ATP levels in the brain while reducing the lactate levels after ischemic injury [14]. However, there is evidence that the use of L-carnitine in ischemic conditions may have negative consequences. The oxidation of fatty acids is not only the most efficient but also the most oxygen-consuming method of energy production, which is fully realized only in the presence of sufficient oxygen under normoxic conditions. During ischemic processes, activated forms of free fatty acids may start to accumulate in cells, blocking the alternative pathway of energy production through glucose oxidation and other substrates [15]. This primarily pertains to the ischemic heart, but the negative effects of long-term L-carnitine therapy have also been demonstrated in the brain [16].

There is an alternative therapeutic approach in which the biosynthesis of L-carnitine is inhibited to prevent the accumulation of acylcarnitines in ischemic tissues. One such drug is mildronate, a competitive inhibitor of γ-butyrobetaine hydroxylase, which catalyzes the conversion of endogenous γ-butyrobetaine to L-carnitine. Mildronate inhibits carnitine biosynthesis to prevent the accumulation of toxic intermediates of fatty acid oxidation in ischemic tissues and compensatorily activates the process of glucose oxidation. Due to these properties, mildronate is effective in the treatment of heart ischemia and its consequences [17]. However, it has also been found to have a beneficial effect on cerebral blood flow and the functionality of the central nervous system (CNS) [18]. The mechanisms of action of mildronate on the CNS are not yet fully understood, but it is suggested that it acts through the biosynthesis of nitric oxide (NO) [19,20]. Although the neuroprotective effect of mildronate has previously been demonstrated in ischemic and neurodegenerative brain diseases, only a single study in 2019 was conducted to investigate its use in the therapy of TBI, which demonstrated its anti-edema, anti-inflammatory, and antioxidant activities [21].

The primary aim of this study was to compare the efficacy of L-carnitine and mildronate injections during the therapeutic window and in the subsequent three days, with the goal of mitigating secondary brain injuries and mitochondrial dysfunctions. However, modeling TBI in rodents presents a challenge due to the need for preliminary craniotomy. This procedure itself may cause certain injuries and trigger a cascade of secondary damage [22]. Therefore, we initially assessed the severity of dysfunctions caused by craniotomy alone, as well as craniotomy followed by TBI, compared to intact animals.

## 2. Results

### 2.1. Changes in Integrity and Quantity of mtDNA in the Brain Following TBI

**Figure 1:**
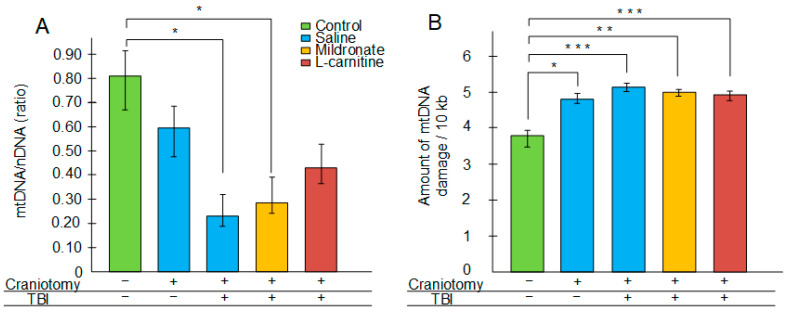
The effect of craniotomy and TBI, as well as injections of metabolic modulators, on the normalized mtDNA copy number (**A**) and the amount of mtDNA damage (**B**) in the injured region of the brain. Differences are statistically significant: * *p* < 0.05, ** *p* < 0.01, *** *p* < 0.001 (Kruskal–Wallis test).

In the injured area of the cortex, a significant decrease in the copy number of mtDNA was observed three days after TBI. In mice that received injections of saline, the mtDNA copy number was reduced by 3.5-fold (*p* < 0.05). In mice that received injections of mildronate, the mtDNA copy number decreased by 8.5-fold (*p* < 0.05). In contrast, no statistically significant reduction in the mtDNA copy number was detected in the group of mice receiving L-carnitine injections three days after TBI (Figure 1A).

The craniotomy procedure resulted in an increase in mtDNA damage levels (+26% compared to control, *p* < 0.05). TBI exacerbated mtDNA damage (+35% compared to control, *p* < 0.001). Injections of mildronate and L-carnitine did not affect the level of mtDNA damage in the context of TBI (+30% compared to control, *p* < 0.01 for the TBI + mildronate group, *p* < 0.001 for the TBI + L-carnitine group) (Figure 1B).

### 2.2. The Effect of TBI and Metabolic Modulators on the Level of Diene Conjugates in the Brain

**Figure 2:**
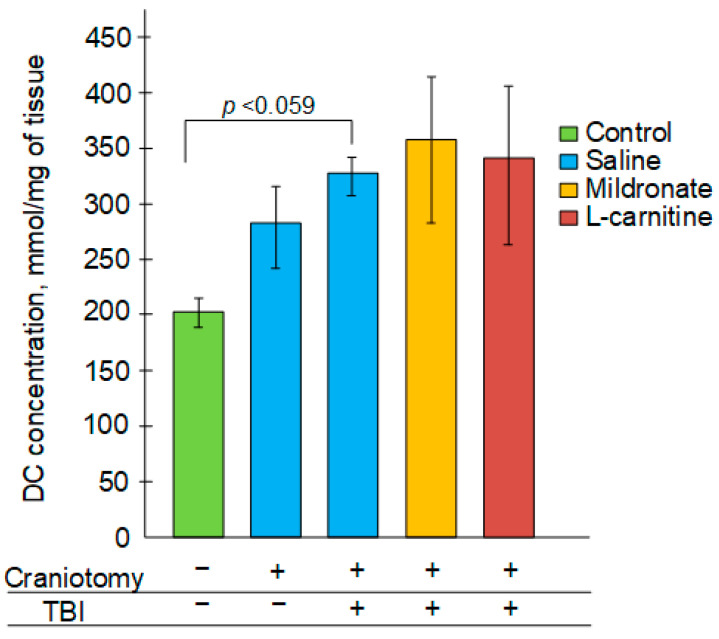
The effect of craniotomy and TB, as well as injections of metabolic modulators, on the concentration of diene conjugates in the injured region of the brains of mice.

TBI resulted in a 64% increase in the levels of primary products of lipid peroxidation—diene conjugates—compared to the control group; however, the differences were not statistically significant (*p* = 0.059). In the groups of mice receiving injections of mildronate and L-carnitine, there was also a trend toward increased levels of diene conjugates, with increases of 76% and 68%, respectively, but these differences were statistically insignificant (Figure 2).

### 2.3. The Effect of TBI and Metabolic Modulators on Gene Expression in the Brain

**Figure 3:**
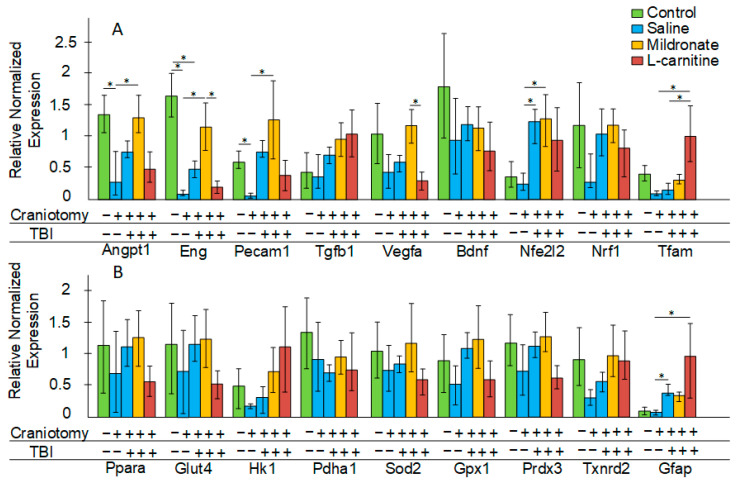
The effect of craniotomy and TBI, as well as injections of metabolic modulators, on the expression of genes involved in the regulation of angiogenesis, neurogenesis, and mitochondrial biogenesis (**A**) and the regulation of glucose metabolism, antioxidant defense, and glial activation (**B**). Differences are statistically significant: * *p* < 0.05 (Kruskal–Wallis test).

The expression of genes involved in angiogenesis was analyzed. Craniotomy resulted in a threefold decrease in angiopoietin 1 (*Angpt1*) expression, a twelvefold decrease in endoglin (*Eng*) expression, and a sixfold decrease in platelet/endothelial cell adhesion molecule 1 (*Pecam*) expression (all *p* < 0.05). TBI caused a threefold reduction in *Eng* expression (*p* < 0.05). Injections of mildronate prevented the decrease in the expression of these genes in the injured region of the brain. Furthermore, in mice receiving mildronate after TBI, vascular endothelial growth factor A (*Vegfa* )expression was found to be twice as high compared to in mice that received L-carnitine injections after TBI (*p* < 0.05). No statistically significant differences were observed in transforming growth factor beta 1 (*Tgfb1*) expression; however, there was a trend toward its increase in all groups subjected to TBI. No changes were noted in brain-derived neurotrophic factor (*Bdnf*) expression. In all TBI-exposed groups, an increase in nuclear factor erythroid 2-like 2 (*Nfe2l2*) expression was observed, but this increase in the brains of mice receiving L-carnitine injections was not statistically significant. We found no difference in the expression of the mitochondrial biogenesis marker nuclear respiratory factor 1 (*Nrf1*), but an increase in mitochondrial transcription factor A (*Tfam*) expression was noted (*p* < 0.05) in the group of mice receiving L-carnitine after TBI. The *Tfam* product is responsible for the regulation of mtDNA transcription (Figure 3A).

No changes were observed in the expression of the peroxisome proliferator-activated receptor alpha (*Ppara*) gene, which is an important transcriptional regulator of metabolism. The physical impact on the brain, as well as metabolic modulators, did not affect the expression of the glucose transporter type 4 (*Glut4*), hexokinase-1 (*Hk1*), and pyruvate dehydrogenase E1 component subunit alpha (*Pdha1*) genes, which are involved in glucose transport and metabolism. No changes were detected in the expression of the antioxidant genes superoxide dismutase 2 (*Sod2*), glutathione peroxidase 1 (*Gpx1*), peroxiredoxin 3 (*Prdx3*), and thioredoxin reductase 2 (*Txnrd2*). In mice subjected to TBI as well as in those receiving L-carnitine, an increase in the expression of the glial activation marker gene glial fibrillary acidic protein (*Gfap*) was observed (both *p* < 0.05 compared to mice subjected only to craniotomy). In mice receiving mildronate, no statistically significant increase in *Gfap* expression was observed following TBI (Figure 3B).

### 2.4. The Effect of TBI and Metabolic Modulators on Gene Expression in Blood

**Figure 4:**
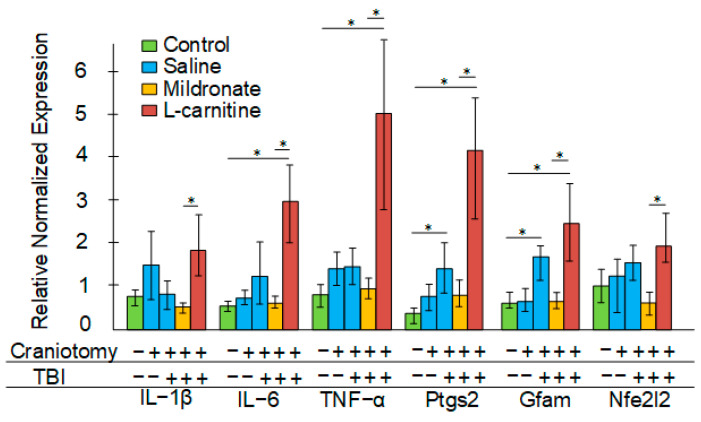
The effect of craniotomy and TBI, as well as injections of metabolic modulators, on gene expression in the blood. Differences are statistically significant: * *p* < 0.05 (Kruskal–Wallis test).

In mice subjected solely to craniotomy, no statistically significant increases in the expression of any of the examined inflammatory markers were observed. Following TBI, the expression of prostaglandin-endoperoxide synthase 2 (*Ptgs2*) and *Gfap* in the blood increased by more than threefold (both *p* < 0.05). The highest expression levels of inflammatory markers such as interleukin 1 beta (*Il1b*), interleukin 6 (*Il6*), tumor necrosis factor (*Tnf*), *Ptgs2*, and *Gfap* were observed in the blood of mice that received L-carnitine injections after TBI. In contrast, in mice receiving mildronate injections, the expression of these genes was significantly lower than in those receiving L-carnitine injections. Furthermore, no increase in the expression of *Ptgs2* and *Gfap* was observed in the blood of this experimental group compared to the control. Additionally, in mice receiving L-carnitine, a significant increase in *Nfe2l2* expression was noted compared to mice receiving mildronate injections (Figure 4).

### 2.5. The Effect of TBI and Metabolic Switching on the Levels of Inflammatory Markers in the Blood

**Figure 5:**
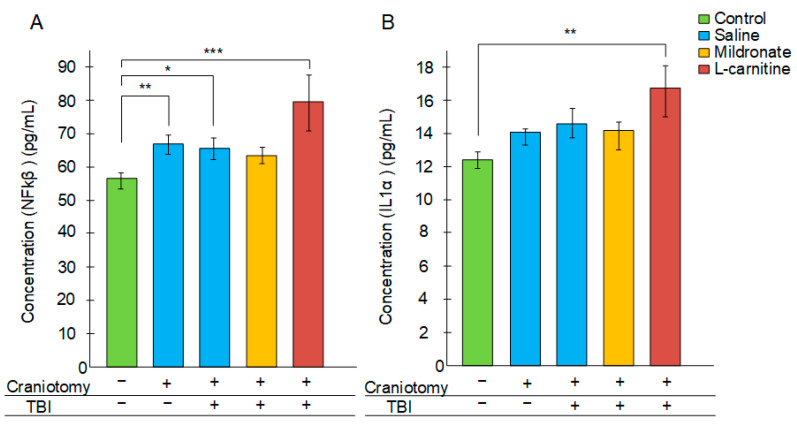
The effect of craniotomy and TBI, as well as injections of metabolic modulators, on the concentration of NFκB (**A**) and IL1a (**B**) in plasma. Differences are statistically significant: * *p* < 0.05, ** *p* < 0.01, *** *p* < 0.001 (Kruskal–Wallis test).

In control mice, the concentration of Nuclear Factor Kappa B (NFκB) in plasma was 56.2 ± 2.4 pg/mL. The craniotomy procedure resulted in a significant increase in NFκB levels to 66.5 ± 2.8 pg/mL (*p* < 0.01). In mice subjected to TBI, an increase in plasma NFκB levels was also observed (64.4 ± 3.2 pg/mL, *p* < 0.05 compared to control). The highest level of NFκB in plasma was found in mice receiving L-carnitine injections after TBI, measuring 78 ± 8.1 pg/mL (*p* < 0.001 compared to control). In mice receiving mildronate, the NFκB level in the blood was 60.9 ± 2.3 pg/mL, and the differences compared to the control were not statistically significant (Figure 5A).

Similarly, the level of interleukin 1 alpha (IL1a) in plasma was highest in mice receiving L-carnitine after TBI, measuring 17.1 ± 1.1 pg/mL compared to 12.7 ± 0.3 in the control group (*p* < 0.01). No statistically significant differences were found when compared to the other experimental groups (Figure 5B).

### 2.6. Changes in the Structure of the Bacterial Composition of the Gut Microbiome

**Figure 6:**
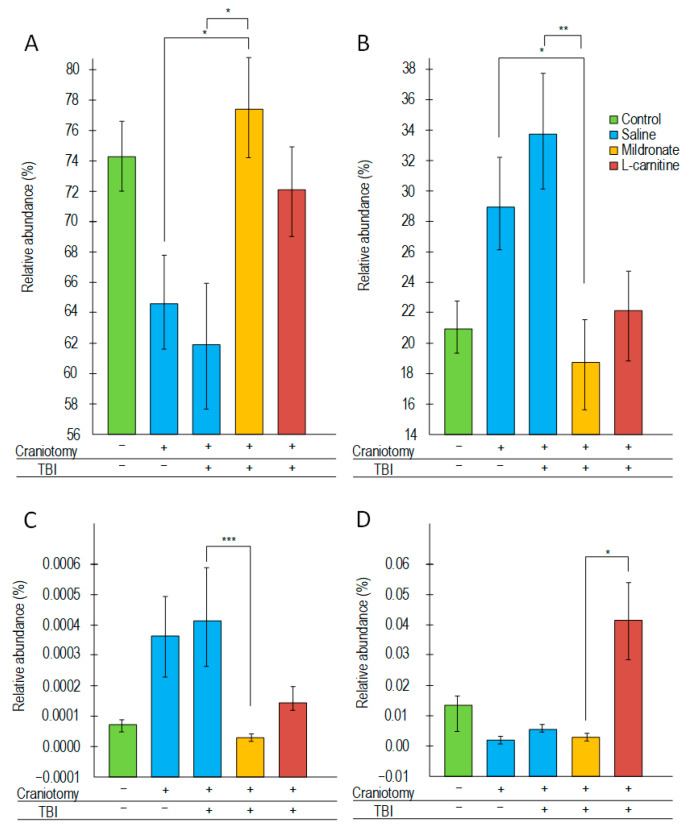
The effect of craniotomy and TBI, as well as injections of metabolic modulators, on the abundance of bacteria from the phylum Bacteroidetes (**A**), Firmicutes (**B**), Deferribacteres (**C**), and Tenericutes (**D**) in the composition of the gut microbiome. Differences are statistically significant: * *p* < 0.05, ** *p* < 0.01, *** *p* < 0.001 (Kruskal–Wallis test).

In all experimental groups, the predominant phyla were *Bacteroidetes* and *Firmicutes*. Mice subjected to craniotomy and TBI showed a trend towards a decrease in the level of *Bacteroidetes* compared to the control, with reductions of 13.1% and 16.9%, respectively. In mice that received mildronate, the content of *Bacteroidetes* increased by 25% compared to mice that underwent only TBI (Figure 6A). Conversely, opposite changes were observed in the phylum *Firmicutes*. In mice subjected to TBI, the level of *Firmicutes* increased from 21% to 33.4%. However, in mice receiving mildronate, the *Firmicutes* level was reduced to 18.6% (*p* < 0.01 compared to mice that underwent TBI). No similar changes were observed in mice receiving L-carnitine (Figure 6B).

No differences were found in the levels of *Actinobacteria, Betaproteobacteria, Delta-* and *Gammaproteobacteria, Epsilonproteobacteria, Candidatus* “Saccharibacteria”, and *Verrucomicrobia*. Mice subjected to craniotomy, as well as those undergoing craniotomy followed by TBI, showed a trend towards an increase in *Deferribacteres*. Their levels were significantly reduced in mice receiving mildronate compared to the group subjected only to TBI (*p* < 0.001) (Figure 6C). Craniotomy and TBI did not have a significant effect on the content of *Tenericutes*, but their numbers increased significantly in the group of mice receiving L-carnitine compared to those receiving mildronate (*p* < 0.05) (Figure 6D).

## 3. Discussion

In experimental modeling of TBI, craniotomy is classically performed beforehand. For this purpose, either a dental burr or a manual trephine is typically used [23]. The control group usually also undergoes craniotomy, which is referred to as a “sham” operation. It was noted that only 4% of the total experimental works related to TBI modeling in ro-dents involved not only sham-operated animals but also completely intact animals that were not subjected to any intervention. It was demonstrated that any craniotomy proce-dure itself (even without a subsequent impact) causes significant brain damage, which is accompanied by substantial cognitive deficits, primarily due to the exacerbation of in-flammatory processes at the site of physical impact on the meninges [22]. We also showed that the craniotomy procedure without subsequent impact leads to an increase in the plasma concentration of the pro-inflammatory cytokine NFκB (Figure 5A). Furthermore, an increase in GFAP expression was noted in both the brain and blood only in the group of mice subjected to TBI but not in those undergoing craniotomy (Figure 3B and Figure 4). This suggests that the level of *Gfap* gene transcript can be considered a reliable marker of inflammation specifically following TBI, rather than merely the disruption of skull integrity. GFAP expression increases upon glial activation due to intensified inflammatory processes; therefore, in 2018, the measurement of GFAP levels in plasma was approved by the FDA as a biomarker for TBI [24].

It is worth noting that after craniotomy, there was a significant reduction in the expression of *Angpt1, Eng,* and *Pecam1* (to a greater extent than after TBI) (Figure 3A). ANGPT1 belongs to the family of endothelial growth factors that function as ligands for the endothelial-specific receptor tyrosine kinase TIE-2. They are considered essential for maintaining the function of the BBB, and early after injury and BBB disruption, a decrease in the levels of ANGPT and TIE-2 proteins is observed [25]. Endoglin (ENG) also plays a crucial role in the regulation of angiogenesis, and its deficiency is associated with the development of hereditary hemorrhagic telangiectasia, as well as an increased risk of hemorrhagic stroke due to vascular fragility [26]. PECAM-1 is classified as a cell adhesion molecule and is involved in maintaining the integrity of blood vessels. The loss of PECAM-1 function leads to impaired BBB function [27], and after transient ischemia, a decrease in *Pecam1* transcripts is observed in the hippocampus, while the protein level, conversely, tends to increase slightly [28]. Thus, we see that craniotomy significantly decreased the expression of genes involved in angiogenesis and the maintenance of BBB functionality. A similarly significant reduction in the expression of *Angpt1* and *Pecam1* was not observed in the group of mice subjected to TBI (Figure 3A). It has previously been shown that after TBI, the expression level of *Pecam1* does not change [29], while the level of ANGPT-1 protein increases only 7–10 days after injury [30].

We measured the amount of mtDNA damage in the brain. It has previously been shown that various neurological disorders, particularly in models of Parkinson’s disease [31,32], ischemic brain injury [33,34], Friedreich’s ataxia [35], HIV-induced neuronal damage [36], and macular degeneration [37], are associated with mtDNA damage. However, the effect of TBI on the integrity of the mitochondrial genome has been less frequently addressed. We demonstrated that an increase in the mtDNA damage levels is observed already with craniotomy (+26% compared to the control). TBI further exacerbated mtDNA damage (+35% compared to control), but no statistically significant differences were found between the sham-operated group and the TBI group (Figure 1B), which suggests that any physical impact on the brain leads to mtDNA damage, and this indicator, in turn, serves as a sensitive marker for such damage.

mtDNA damage can be induced by secondary processes that arise several hours after TBI, with one of the most significant being oxidative stress [38]. In addition to the increased levels of mtDNA damage, we observed an increase in the levels of 4-hydroxyalkenals (4-HNEs) in the brain (Figure 2), which are markers of lipid peroxidation and thus also serve as indicators of oxidative stress [39]. However, TBI, but not craniotomy, was associated with a significant decrease in the mtDNA copy numbers, which may indirectly indicate a reduction in the number of mitochondria. It has previously been shown that repeated mild TBIs lead to a decrease in mitochondrial numbers in nearly all brain regions; thus, the assessment of mtDNA levels in brain regions is considered one of the metrics for evaluating the severity of secondary brain injuries caused by trauma [40]. Moreover, extremely low levels of mtDNA have been noted in combat veterans with diagnosed post-traumatic stress disorder (PTSD) following injuries [41]. Therefore, the potential for modulating mitochondrial biogenesis to increase mtDNA copy numbers may be significant not only in the acute recovery phase following TBI but also for the therapy of the long-term, including psychological, consequences of head trauma.

Thus, we can conclude that the consequences of craniotomy and craniotomy + TBI are generally similar. However, craniotomy appears to induce a stronger suppression of the expression of genes regulating angiogenesis compared to TBI, which likely contributes to BBB damage. The increase in mtDNA damage is observed equally with both craniotomy and TBI. Notably, specifically after TBI, there is a decrease in mtDNA copy numbers, an increase in the levels of lipid peroxidation products, and an elevation of glial activation markers in both the blood and the damaged area of the brain.

Next, we evaluated the effects of metabolic modulators on certain markers of secondary brain injury following TBI. L-carnitine is necessary for the transport of fatty acids into mitochondria, and during stress, it can provide cells with an additional source of energy. It has been shown that the plasma levels of L-carnitine are significantly decreased in patients three days post TBI. The authors attributed this to the increased consumption of free L-carnitine during hypercatabolism after injury, when β-oxidation becomes an important source of energy for the brain [42]. Earlier studies have investigated the neuroprotective effects of L-carnitine in a model of permanent focal ischemia. L-carnitine was administered via an atraumatic catheter in a dosage range from 25 mg/kg to 800 mg/kg. A dose-dependent increase in ATP generation was observed in the concentration range of 50 to 200 mg/kg, while further increases in the drug dosage did not result in the additional enhancement of this parameter [43]. L-carnitine acts as an activator of the Nrf2/ARE signaling pathway [44], which may contribute to the activation of antioxidant defenses and a reduction in oxidative stress in the damaged area of the brain. Indeed, we demonstrated that mice receiving L-carnitine exhibited increased expression of the *Nfe2l2* gene, which encodes Nrf2, but only in plasma (Figure 4). No increase in the expression of either *Nfe2l2* (Figure 3A) or any of the antioxidant genes controlled by the Nrf2/ARE pathway was observed in the brain (Figure 3B).

It has been previously reported that L-carnitine may reduce inflammatory reactions following TBI in a mouse model [45], but these findings are not supported by the results from placebo-controlled clinical trials [12]. In our experiment, on the contrary, the group of mice receiving L-carnitine injections after TBI exhibited the highest levels of inflammatory transcripts (Figure 4) and protein markers in the blood (Figure 5), as well as an increased expression of glial activation markers in the brain (Figure 4). Although L-carnitine is generally considered an anti-inflammatory agent, it can exhibit opposing properties under certain physiological conditions. The addition of the dietary supplement L-carnitine may lead to its conversion to trimethylamine (TMA) by certain bacteria in the microbiome. TMA is then metabolized in the liver to trimethylamine N-oxide (TMAO) [46]. Typically, increased levels of TMAO in plasma are regarded as a predictor of cardiovascular diseases, and one potential mechanism for the toxic effects of TMAO is related to its pro-inflammatory effects [47]. Among the components of the bacterial microbiota commonly associated with the conversion of L-carnitine to TMA (and consequently to TMAO), bacteria from the phylum *Tenericutes* are typically mentioned [48], along with certain genera from the phyla *Firmicutes* and *Proteobacteria* [49]. We found that in the TBI + L-carnitine group of mice, there was a significant increase in the abundance of *Tenericutes* (Figure 6). In mice receiving the L-carnitine antagonist meldonium, the abundance of *Firmicutes* was reduced, while the proportion of another dominant phylum, *Bacteroidetes*, increased; however, such changes were not observed in the structure of the bacterial community of the intestinal microbiome of mice receiving L-carnitine (Figure 6A,B). Overall, following TBI, the administration of L-carnitine induces certain alterations in the intestinal microbiota that may facilitate the conversion of L-carnitine to TMA and subsequently to TMAO, potentially mediating the observed inflammatory processes in this group of mice.

However, L-carnitine also exhibited positive effects. We showed that in mice receiving L-carnitine, there was no statistically significant decrease in the mtDNA copy numbers following TBI (Figure 1A). This coincided with the observation of the maximum expression of the *Tfam* gene in this experimental group of mice (Figure 3A), which may overall indicate the activation of mitochondrial biogenesis. It has been previously demonstrated that the dietary supplement L-carnitine can stimulate mitochondrial biogenesis in the brain via a TFAM-dependent mechanism in aged rats [50].

Mildronate is an antagonist of L-carnitine, which promotes a shift in metabolism towards glucose oxidation. This is particularly important under conditions of oxygen deficiency caused by the disruption of blood supply to the damaged area of the brain. There is evidence supporting a NO-dependent action for mildronate. Experimental studies have shown that the administration of mildronate induced an increase in the concentration of γ-butyrobetaine esters because mildronate inhibits the hydroxylation of γ-butyrobetaine to L-carnitine. The resulting γ-butyrobetaine esters accumulated in the blood, where they bound to their specific receptors and m-acetylcholine receptors. This, in turn, may provoke an increase in nitric oxide synthase (NOS) activity and enhanced NO production [19,20]. The inhalation of NO has been shown to improve cerebral blood flow, reduce lesion volume, decrease brain edema, and mitigate BBB damage following TBI [51]. Elevated levels of NO may facilitate angiogenesis via the VEGF-dependent pathway [52]. We observed that mice receiving mildronate injections after TBI exhibited an increased expression of *Vegfa* compared to those receiving L-carnitine. Furthermore, this group of mice did not show a TBI-induced reduction in the expression of *Angpt1*, *Eng*, or *Pecam1* (Figure 3A). It is known that PECAM-1 can form a complex with eNOS and regulate NO production [53]. Thus, we can hypothesize that mildronate’s involvement in NO metabolism may mediate its effects on angiogenesis in the context of TBI, contributing to improvements in cerebral blood flow and supporting BBB functionality in the first days following the injury.

The anti-inflammatory effect of mildronate has been previously demonstrated. In mice that received mildronate after TBI, there was a reduction in myeloperoxidase activity, which is one of the markers of inflammation [21]. In this study, we found that mildronate prevented a statistically significant increase in the level of *Gfap* transcript in the brain (Figure 3B) and NFκB in plasma (Figure 5A). Moreover, the expression levels of key inflammatory markers in the blood were comparable to those in control mice (Figure 4). Mice receiving mildronate exhibited an increase in the abundance of Bacteroidetes, while the abundance of *Firmicutes* was decreased (Figure 6A,B). This ratio can also be considered an anti-inflammatory factor. It has been previously shown that the *Bacteroidetes*/*Firmicutes* ratio negatively correlates (r_s_ = −0.41, *p* = 0.03) with C-reactive protein levels in obesity. Fecal calprotectin has also been found only in obese patients with a decreased *Bacteroidetes*/*Firmicutes* ratio [54]. We observed that in mice receiving mildronate, the level of *Deferribacteres* was reduced compared to in mice subjected only to TBI (Figure 6B). This phylum of bacteria is also typically associated with inflammatory processes in the intestine. It has been shown that increased levels of *Deferribacteres* positively correlate with the levels of pro-inflammatory cytokines IL-1β, IL-6, IL-18, and TNF-α [55]. Thus, unlike L-carnitine, mildronate exerts a distinct anti-inflammatory effect, which, in conjunction with its ability to influence the expression of genes associated with blood vessel growth, positions mildronate as a promising compound for mitigating the secondary consequences of TBI.

## 4. Materials and Methods

### 4.1. Animals and Experimental Design

In the experiment, male mice of the C57BL/6 strain, aged 2 months, were used, which were obtained from the “Stolbovaya nursery” (Russia, Moscow region). All experiments conducted with the animals were reviewed and approved by the Ethics Committee for Biomedical Research at the Voronezh State University (protocol No. 42-03 dated October 14, 2024). The mice were kept under standard conditions: temperature = 25 °C, a 12 h light cycle, and a relative humidity of at least 40%.

**Figure 7:**
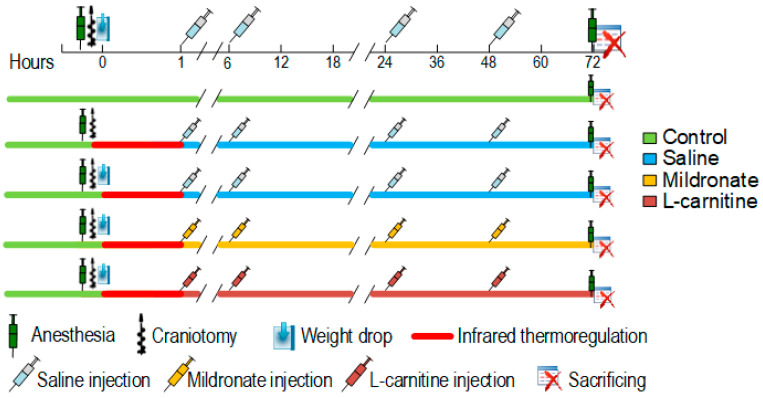
Timeline of the experiment.

Mice were divided into five groups. The first group (control, *n* = 10) included intact mice that underwent no procedures. Mice in the second group (sham group, or craniotomy group, *n* = 9) were placed under isoflurane anesthesia (1.5% in air), followed by a skin incision and craniotomy. 1 h, 6 h, 24 h, and 48 h after the procedure, the mice were administered intraperitoneal injections of saline. The third group of mice (TBI + saline, *n* = 10) underwent TBI following anesthesia and craniotomy. Similarly, 1 h, 6 h, 24 h, and 48 h post operation, these mice received intraperitoneal injections of saline. The fourth group of mice (TBI + mildronate, *n* = 9) was subjected to TBI as well. After the same time intervals (1 h, 6 h, 24 h, and 48 h post operation), mice in this group received intraperitoneal injections of mildronate (Grindex, Riga, Latvia) at a dose of 100 mg/kg. The fifth group of mice (TBI + L-carnitine, *n* = 10) also experienced TBI. At the aforementioned time points, these mice were administered intraperitoneal injections of L-carnitine (KorolevPharm, Korolev, Russia) at a dose of 100 mg/kg. The concentration of both drugs was selected based on previous studies [56,57]. Following a 72 h period post operation, fecal samples from the mice were collected, and the mice were euthanized. The experimental timeline is presented in Figure 7. Prior to euthanasia, blood samples were collected from the mice via retro-orbital sinus puncture and were immediately separated into plasma and cellular components. For this procedure, whole blood was centrifuged at 3500× *g* for 5 min.

### 4.2. Modeling of TBI

Mice were anesthetized using isoflurane (1.5% in air). Subsequently, the heads of the mice were shaved, the skin was incised along the sagittal suture, and a craniotomy was performed by drilling a 4 mm diameter hole in the skull using a dental burr (JessNail DM206, Hong Kong, China). This hole was specifically located to access the sensorimotor cortex, which is situated lateral to the sagittal suture and caudal to the bregma. TBI was induced using a stereotaxic impactor, the 68099II Precise Impactor (RWD, Nanshan, China). The impact was delivered to the right hemisphere using a striker with a diameter of 3 mm, and the impact depth was set to 1.2 mm. Afterward, sutures were applied with a surgical needle, and the wounds were treated with fukorcin. Postoperatively, rectal temperature was monitored using a multimeter and maintained within the range of 36–37 °C by heating the mice with infrared heat lamps.

### 4.3. Measurement of Gene Expression

Total RNA was isolated from the injured brain region at 72 h post TBI and from whole blood using the ExtractRNA kit (Evrogen, Moscow, Russia). Reverse transcription was performed using the “REVERTA-L” kit (AmpliSens, Moscow, Russia). The resulting cDNA was used to assess gene expression levels through quantitative PCR employing the Bio-Rad CFX96TM Real-Time System (Bio-Rad, Hercules, CA, USA) and the 5X qPCRmix-HS SYBR master mix (Evrogen, Moscow, Russia). The primer sequences are presented in Table 1. The reaction conditions were as follows: initial denaturation at 95 °C for 3 min, followed by 40 cycles of 20 s at 95 °C, 30 s at 59 °C, and 30 s at 72 °C. The raw Cq values and representative examples of PCR product accumulation curves are provided in the Table S1 and Figures S1–S3.

### 4.4. Study of mtDNA Quality Control

Total DNA was isolated from samples using the DNA-Sorb kit (AmpliSens, Moscow, Russia). The relative quantity of mtDNA was evaluated using real-time PCR. The reaction was conducted using the 5X qPCRmix-HS SYBR master mix (Evrogen, Moscow, Russia). The following primer pair was used for the amplification of mouse mtDNA: forward: 5′-ACGAGGGTCCAACTGTCTCTTA-3′; reverse: 5′-AGCTCCATAGGGTCTTCTCGT-3′. The amplification of the nuclear DNA segment used for normalization was performed with the following primers: forward: 5′-GGCTCCCTAGGCCCCTCCTG-3′; reverse: 5′-TCCCAACTCGGCCCCCAACA-3′. The reaction conditions were as follows: initial denaturation at 95 °C for 3 min, followed by 40 cycles of 20 s at 95 °C, 30 s at 59 °C, and 30 s at 72 °C.

The amount of mtDNA damage was evaluated using real-time long-range PCR with an Encyclo polymerase kit (Evrogen, Moscow, Russia). The method is based on the assumption that the presence of damage in the DNA structure, such as double- and single-strand breaks, modified bases, or their adducts, will hinder the activity of DNA polymerase and decrease the efficiency of the reaction [7]. In addition to amplifying long (~2000 bp) products, short (~100 bp) fragments were simultaneously amplified to normalize the level of damage per copy of mtDNA in the studied sample. The primer sequences are presented in Table 2. The reaction conditions were as follows: initial denaturation at 95 °C for 3 min, followed by 30 cycles of 20 s at 95 °C, 30 s at 59 °C, and 4 min 30 s at 72 °C. The amount of additional damage in mtDNA was calculated per 10 kb using the following Formula (1):

(1) $$D=\left(1-2^{-(\Delta cq\;L-\Delta cq\;S)}\right)*\;\frac{10000\;bp}{Fragment\;lenght}$$

### 4.5. Assessment of the Level of Diene Conjugates

Damaged cortical fragments were homogenized in phosphate buffer, and then the homogenate was centrifuged for 5 min at 500× *g*. A total of 125 μL of the supernatant was collected and mixed with an equal volume of saline, 1.5 mL of heptane, and 1.5 mL of isopropyl alcohol. The mixture was centrifuged for 10 min at 3000× *g*. To the supernatant, 1/10 of the volume of distilled water was added, and it was transferred to new tubes, where phase separation was observed after vigorous shaking. The heptane phase was carefully collected into a new tube, and 500 μL of ethanol was added. Measurements were conducted using a Hitachi U-2900 spectrophotometer (Hitachi, Japan) at a wavelength of 233 nm. The concentration of diene conjugates in the homogenate was calculated using the following Formula (2):

(2) $$C_{dc}=\frac{V_{total}\times D\times 10^{6}}{L\times E\times m\times V_{add}}$$

where *C_dc_* is the concentration of diene conjugates (mmol/g); *V_total_* is the volume of the obtained sample (ml); *D* is the optical density (units); *L* is the length of the optical path (1 cm); *E* is the molar extinction coefficient equal to 2.2 × 10^5^ M^−1^ s^−1^; m is the mass of the brain fragment; and *V_add_* is the volume of the inserted sample (ml).

### 4.6. Measurement of Pro-Inflammatory Cytokines

The levels of inflammatory markers in the plasma of mice were assessed using the Bio-Rad iMark microplate absorbance readerplate (Bio-Rad, Hercules, CA, USA). ELISA kits for NFκB (Cat. no. SEB824Mu) and IL1a (Cat. no. SEA071Mu) (both Cloud-Clone Corp., Houston, TX, USA) were utilized according to the manufacturer’s protocol.

### 4.7. Assessment of the Bacterial Composition of the Intestinal Microbiome

Total DNA from mouse feces was extracted using the DNA-Sorb kit (AmpliSens, Moscow, Russia). Phylum-specific primers were used to amplify fragments of bacterial 16S rRNA, Table 3 [58]. The reaction was conducted using the 5X qPCRmix-HS SYBR master mix (Evrogen, Moscow, Russia). The reaction conditions were as follows: initial denaturation at 95 °C for 3 min, followed by 40 cycles of 10 s at 95 °C, 10 s at 55 °C, and 15 s at 72 °C. The quantitative ratio of each phylum was calculated using the following Formula (3):

(3) $$Number\;of\;bacteria=\frac{{E_{universal}}^{{Cq}_{universal}}}{{E_{specific}}^{{Cq}_{specific}}}\times 100$$

where *E* is the efficiency of PCR.

### 4.8. Statistical Analysis

Statistical analysis of the data was performed using the STATISTICA 12 software package (StatSoft, Tulsa, OK, USA). Results are presented as mean values ± standard error of the mean (SEM). To assess the significance of differences between groups, the non-parametric Kruskal–Wallis test was employed. The statistical significance level was set at *p* < 0.05.

## 5. Conclusions

Both craniotomy and the subsequent impact to the brain initiate a cascade of secondary injuries. Craniotomy significantly reduces the expression of genes responsible for angiogenesis, as well as causing damage to mtDNA and increasing the level of the pro-inflammatory factor NFκB in plasma. The subsequent TBI exacerbates inflammatory processes, leads to glial activation, and contributes to a reduction in the mtDNA content in the damaged region of the brain. Distinct approaches to modulating metabolism after TBI elicit different protective effects. L-carnitine stimulates mitochondrial biogenesis and maintains mitochondrial mass in the damaged area of the brain; however, it appears that L-carnitine itself is metabolized to TMAO, which increases the intensity of inflammatory processes. Mildronate, which inhibits the synthesis and transport of L-carnitine into cells, on the other hand, reduces systemic and local inflammation in the damaged area of the brain and improves angiogenesis but does not have a positive impact on the integrity or the copy number of mtDNA. A combined therapy of mildronate and L-carnitine may represent the most promising approach to TBI treatment, although this issue requires further investigation.

## Figures and Tables

**Table 1:** Primer sequences for measurement of gene expression.

Gene	Forward Primer 5′-3′	Reverse Primer 5′-3′
*18s*	CGGCTACCACATCCAAGGAA	GCTGGAATTACTGTGGCT
*Angpt1*	AGCAATCCTTAGCATAGGGGC	TGTGTAACCGTTCAGCGTGG
*Bdnf*	AAGGACGCGGACTTGTACAC	CGCTAATACTGTCACACACGC
*Eng*	CAACTTAGCTCTGCGCCCTA	GGTGGAGGCTTGGGATACTC
*Gapdh*	GGCTCCCTAGGCCCCTCCTG	TCCCAACTCGGCCCCCAACA
*Gfap*	CGAAGAAAACCGCATCACCA	CCGCATCTCCACCGTCTTTA
*Glut4*	CCTCCCGCCCTTAGTTG	CTGCAAAGCGTAGGTACCA
*Gpx1*	AGTCCACCGTGTATGCCTTC	GTGTCCGAACTGATTGCACG
*Hk1*	GTTCGAGAAGATGGTGAGCG	AGAGTTCCCATCCCGTTTCA
*Nfe2l2*	CTCTCTGAACTCCTGGACGG	GGGTCTCCGTAAATGGAAG
*Nrf1*	AGCACGGAGTGACCCAAA	TGTACGTGGCTACATGGACCT
*Pdha1*	GTTTTGGGCGTGGCTTCG	GGCTTGCCGGCTTCTG
*Pecam1*	GGAACGAGAGCCACAGAGAC	TTCCATTAAGGGAGCCTTCCG
*Ppara*	AGAGCCCCATCTGTCCTCTC	ACTGGTAGTCTGCAAAACCAAA
*Prdx3*	GGTTGCTCGTCATGCAAGTG	CCACAGTATGTCTGTCAAACA
*Sod2*	CAGACCTGCCTTACGACTATGG	CTCGGTGGCGTTGAGATTGTT
*Tfam*	ATTCCGAAGTGTTTTTCCAGCA	TCTGAAAGTTTTCGATCTGGGT
*Tgfb1*	CATGACATGAACCGGCCCTT	GAAGTTGGCATGGTAGCCCT
*Txnrd2*	GATCTCTTGGTGATCGGTGGG	CGGGGAGAGGGTTCCACATA
*Vegfa*	TATTCAGCGGACTCACCAGC	AACCAACCTCCTCAAACCGT
*Angpt1*	AGCAATCCTTAGCATAGGGGC	TGTGTAACCGTTCAGCGTGG
*Bdnf*	AAGGACGCGGACTTGTACAC	CGCTAATACTGTCACACACGC
*Eng*	CAACTTAGCTCTGCGCCCTA	GGTGGAGGCTTGGGATACTC

**Table 3:** Primer sequences for assessment of the bacterial composition of the intestinal microbiome.

Group	ID Primers	The Nucleotide Sequence (5′-3′)
*Bacteroidetes*	Bac960F	GTTTAATTCGATGATACGCGAG
	Bac1100R	TTAASCCGACACCTCACGG
*Firmicutes*	Firm934F	GGAGYATGTGGTTTAATTCGAAGCA
	Firm1060R	AGCTGACGACAACCATGCAC
*Actinobacteria*	Act664F	TGTAGCGGTGGAATGCGC
	Act941R	AATTAAGCCACATGCTCCGCT
*Betaproteobacteria*	Beta979F	AACGCGAAAAACCTTACCTACC
	Beta1130R	TGCCCTTTCGTAGCAACTAGTG
*Gammaproteobacteria*	Gamma877F	GCTAACGCATTAAGTRYCCCG
	Gamma1066 R	GCCATGCRGCACCTGTCT
*Epsilonproteobacteria*	Epsilon940F	TAGGCTTGACATTGATAGAATC
	Epsilon1129 R	CTTACGAAGGCAGTCTCCTTA
*Deferribacteres*	Defer1115F	CTATTTCCAGTTGCTAACGG
	Defer1265R	GAGHTGCTTCCCTCTGATTATG
*Saccharibacteria*	Sac1031F	AAGAGAACTGTGCCTTCGG
	Sac1218R	GCGTAAGGGAAATACTGACC
*Tenericutes*	Ten662F	ATGTGTAGCGGTAAAATGCGTAA
	Ten862R	CMTACTTGCGTACGTACTACT
*Verrucomicrobia*	Ver1165F	TCAKGTCAGTATGGCCCTTAT
	Ver1263R	CAGTTTTYAGGATTTCCTCCGCC
*Universal*	926F	AAACTCAAAKGAATTGACGG
	1062R	CTCACRRCACGAGCTGAC

**Table 2:** Primer sequences for assessing the amount of mtDNA damage.

ID Primer	Forward Primer 5′-3′	ID Primer	Reverse Primer 5′-3′
ChrM: Pr. 1	TAAATTTCGTGCCAGCCACC	ChrM: Pr. 1 (long)	ATGCTACCTTTGCACGGTCA
ChrM: Pr. 2	ACGAGGGTCCAACTGTCTCTTA	ChrM: Pr. 2 (short)	AGCTCCATAGGGTCTTCTCGT
		ChrM: Pr. 2 (long)	CCGGCTGCGTATTCTACGTT
ChrM: Pr. 3	CTAGCAGAAACAAACCGGGC	ChrM: Pr. 3 (long)	TTAGGGCTTTGAAGGCTCGC
ChrM: Pr. 7	TCATTCTTCTACTATCCCCAATCC	ChrM: Pr. 7 (long)	TGGTTTGGGAGATTGGTTGATG
ChrM: Pr. 8	CCCCAATCCCTCCTTCCAAC	ChrM: Pr. 8 (long)	GGTGGGGAGTAGCTCCTTCTT
ChrM: Pr. 9	AAGAAGGAGCTACTCCCCACC	ChrM: Pr. 9 (long)	GTTGACACGTTTTACGCCGA

## Data Availability

The data generated and analyzed during the current study are available from the corresponding author on reasonable request.

## Supplementary Materials

The following supporting information can be downloaded at https://www.mdpi.com/article/10.3390/ijms26072902/s1.

## Abbreviations

The following abbreviations are used in this manuscript:

TBI	Traumatic brain injury
mtDNA	Mitochondrial DNA
BBB	Blood–brain barrier
ROS	Reactive oxygen species
CNS	Central nervous system
NO	Nitric oxide
PTSD	Post-traumatic stress disorder
TMA	Trimethylamine
TMAO	Trimethylamine N-oxide
NOS	Nitric oxide synthase
NFκB	Nuclear Factor Kappa B
*Angpt1*	Angiopoietin 1
*Eng*	Endoglin
*Pecam*	Platelet/endothelial cell adhesion molecule 1
*Vegfa*	Vascular endothelial growth factor A
*Tgfb1*	Transforming growth factor beta 1
*Bdnf*	Brain-derived neurotrophic factor
*Nfe2l2*	Nuclear factor erythroid 2-like 2
*Nrf1*	Nuclear respiratory factor 1
*Tfam*	Transcription factor A
*Ppara*	Peroxisome proliferator-activated receptor alpha
*Glut4*	Glucose transporter type 4
*Hk1*	Hexokinase-1
*Pdha1*	Pyruvate dehydrogenase E1 component subunit alpha
*Sod2*	Superoxide dismutase 2
*Gpx1*	Glutathione peroxidase 1
*Prdx3*	Peroxiredoxin 3
*Txnrd2*	Thioredoxin reductase 2
*Gfap*	Glial fibrillary acidic protein
*Ptgs2*	Prostaglandin-endoperoxide synthase 2
*Il1b*	Interleukin 1 beta
*Il6*	Interleukin 6
*Tnf*	Tumor necrosis factor
