# Supplementary material for: L-Carnitine and Mildronate Demonstrate Divergent Protective Effects on Mitochondrial DNA Quality Control and Inflammation Following Traumatic Brain Injury
Source: Int J Mol Sci. 2025 Mar 22;26(7):2902. doi: 10.3390/ijms26072902 (PMC11988827; doi:10.3390/ijms26072902)
Supplement: Supplementary file 1 [file ijms-26-02902-s001.zip › ijms-3508487-supplementary.pdf]

L-Carnitine and Mildronate Demonstrate Divergent Protective Effects on Mitochondrial DNA Quality Control and Inflammation Following Traumatic Brain Injury

Artem P. Gureev [1], Veronika V. Nesterova [1], Polina I. Babenkova [1], Mikhail E. Ivanov [2], Egor Y. Plotnikov [2] and Denis N. Silachev [2*]

Table S1.

Number of mtDNA damage and mtDNA copy number

Fragment	Control	Craniotomy+saline	TBI+saline	TBI+mildronate	TBI+L-carnitine
18 s (nucl)	17.22±0.377	17.52±0.513	17.46±0.312	17.17±0.353	17.74±0.407
Gapdh (nucl)	16.50±0.279	17.02±0.3	17.55±0.610	17.23±0.266	17.24±0.550
Short (mito)	12.82±0.216	13.79±0.453	14.46±0.527	14.55±0.469	14.35±0.544
1 long (mito)	11.88±0.388	13.38±0.454	14.90±0.983	14.40±0.586	15.12±0.967
2 long (mito)	11.38±0.204	12.58±0.553	14.03±1.215	13.53±0.570	13.23±0.543
3 long (mito)	11.43±0.283	13.21±0.553	14.25±0.610	14.16±0.490	14.26±0.594
7 long (mito)	11.77±0.299	14.10±0.537	14.89±0.805	14.77±0.650	13.91±1.053
8 long (mito)	11.17±0.177	13.12±0.609	14.11±0.744	13.94±0.651	13.80±0.760
9 long (mito)	11.12±0.231	12.75±0.542	13.84±0.771	13.57±0.533	13.50±0.671

Gut microbiome

Filum	Control	Craniotomy+saline	TBI+saline	TBI+mildronate	TBI+L-carnitine
Universal	13.521±0.430	15.062±0.842	15.746±0.637	13.496±0.549	14.173±0.703
Bacteroidetes	13.889±0.348	15.423±0.902	16.44±0.690	13.354±0.693	14.409±0.907
Firmicutes	14.953±0.412	15.658±0.649	16.257±0.674	14.919±0.3	15.401±0.704
Actinobacteria	23.38±0.685	24.131±0.605	25.237±0.945	24.091±0.630	24.019±0.644
Betaproteobacteria	21.271±0.650	22.394±0.386	22.8±0.415	22.111±0.515	22.372±0.433
Gammaproteobacteria	30.389±1.229	30.016±1.111	30.298±0.875	31.252±1.842	31.047±1.265
Epsilonproteobacteria	20.414±0.776	20.589±0.917	22.678±0.810	21.545±1.189	20.776±0.988
Deferribacteres	36.948±1.112	37.47±1.305	36.809±0.864	39.086±1.305	37.534±1.001
Saccharibacteria	27.751±2.947	25.803±2.497	24.549±0.780	25.401±2.588	24.446±2.499
Tenericutes	30.048±1.890	31.744±0.622	32.106±1.103	32.447±1.932	28.779±2.373
Verrucomicrobia	31.544±1	28.511±1.126	33.163±0.985	29.18±2.665	28.081±1.826

Gene expression (brain)

Gene	Control	Craniotomy+saline	TBI+saline	TBI+mildronate	TBI+L-carnitine
Gapdh	22.09±0.237	20.97±0.454	21.91±0.250	21.54±0.413	21.08±0.361
18s	16.95±0.193	16.61±0.28	16.87±0.269	16.48±0.429	16.44±0.512
Bdnf	28.26±0.643	28.41±0.862	28.6±0.238	28.34±0.322	28.44±0.525
Angpt	26.02±0.283	27.34±1.643	26.71±0.196	25.59±0.130	26.61±0.607
Eng	35.54±0.264	38.46±2.538	37.11±1.149	35.43±0.302	37.6±1.614
Pecam1	32.88±0.315	34.61±1.28	32.51±0.310	31.34±0.655	32.77±1.244

Gfap	31.09±0.675	31.13±0.55	28.66±0.620	28.34±0.580	26.8±0.92
Glut4	28.36±0.837	28.46±1.117	28.32±0.300	27.84±0.493	28.77±0.749
Gpx1	30.87±0.803	30.74±0.882	30.35±0.354	29.8±0.599	30.43±0.7326
Hk1	36.67±1.086	37.22±0.586	36.91±1.234	35.67±0.663	34.84±0.887
Nfe2l2	29.68±1.553	29.3±1.492	27.77±0.286	27.35±0.372	27.47±0.687
Nrf1	31.87±0.847	33.01±2.028	31.87±0.467	31.35±0.350	31.62±0.787
Pdha1	33.73±0.590	33.44±0.868	34.24±0.367	33.26±0.35	33.43±0.724
Ppara	32.91±0.913	32.86±1.33	32.78±0.41	32.26±0.443	33.16±0.767
Prdx3	28.98±0.468	28.86±0.708	28.95±0.178	28.47±0.29	28.94±0.449
Ptgs2	29.48±0.584	29.51±0.99	29.5±0.282	28.69±0.323	29.1±0.641
Sod2	28.19±0.573	27.88±0.721	28.36±0.279	27.44±0.593	28.17±0.661
Tfam	33.7±0.393	35.54±0.596	35.49±1.28	33.89±0.648	31.65±0.904
Tgfb1	35.11±0.98	34.52±1.100	34.18±0.137	33.39±0.253	33±0.428
Txnrd2	34.6±0.775	35.37±0.435	35±0.480	33.75±0.583	33.62±0.613
Vegfa	26.58±0.711	27.09±0.945	27.15±0.308	25.84±0.332	27.33±0.697

Gene expression (blood)

Gene	Control	Craniotomy+saline	TBI+saline	TBI+mildronate	TBI+L-carnitine
18S	17±1.016	17.26±0.535	16.27±0.46	16.52±0.537	21.1±0.854
Gapdh	24.23±0.78	22.89±0.552	23.08±0.506	23.83±0.417	23.55±0.306
Gfap	33.99±0.47	33.75±0.861	33.26±0.500	34.1±0.550	34.26±0.492
IL-1b	31.2±0.316	29.95±0.7547	30.84±0.674	32.27±0.313	31.69±0.272
IL-6	32.46±0.339	31.37±0.497	31.6±0.748	32.65±0.434	32.14±0.328
Ptgs2	31.96±0.818	30.51±0.504	30.63±0.552	30.84±0.549	30.98±0.31
Tnf	30.33±0.448	29.43±0.442	29.62±0.515	30.32±0.296	30.11±0.401
Nfe2l2	29.94±0.380	29.54±0.898	29.16±0.529	31.04±0.791	31.02±0.461

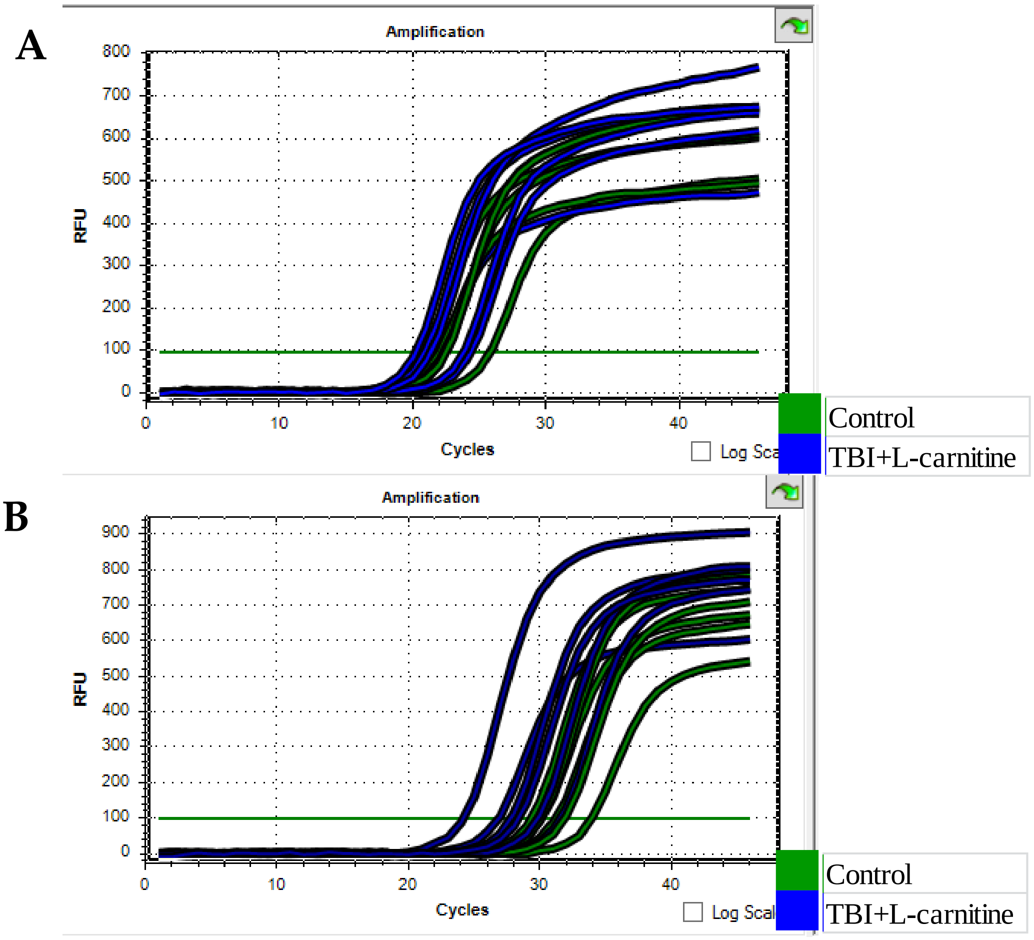

Figure S1. Representative examples of PCR product accumulation curves during gene expression estimation in the Control and TBI + L-carnitine groups. Curves for Gapdh (reference) (A) and Gfap (B).

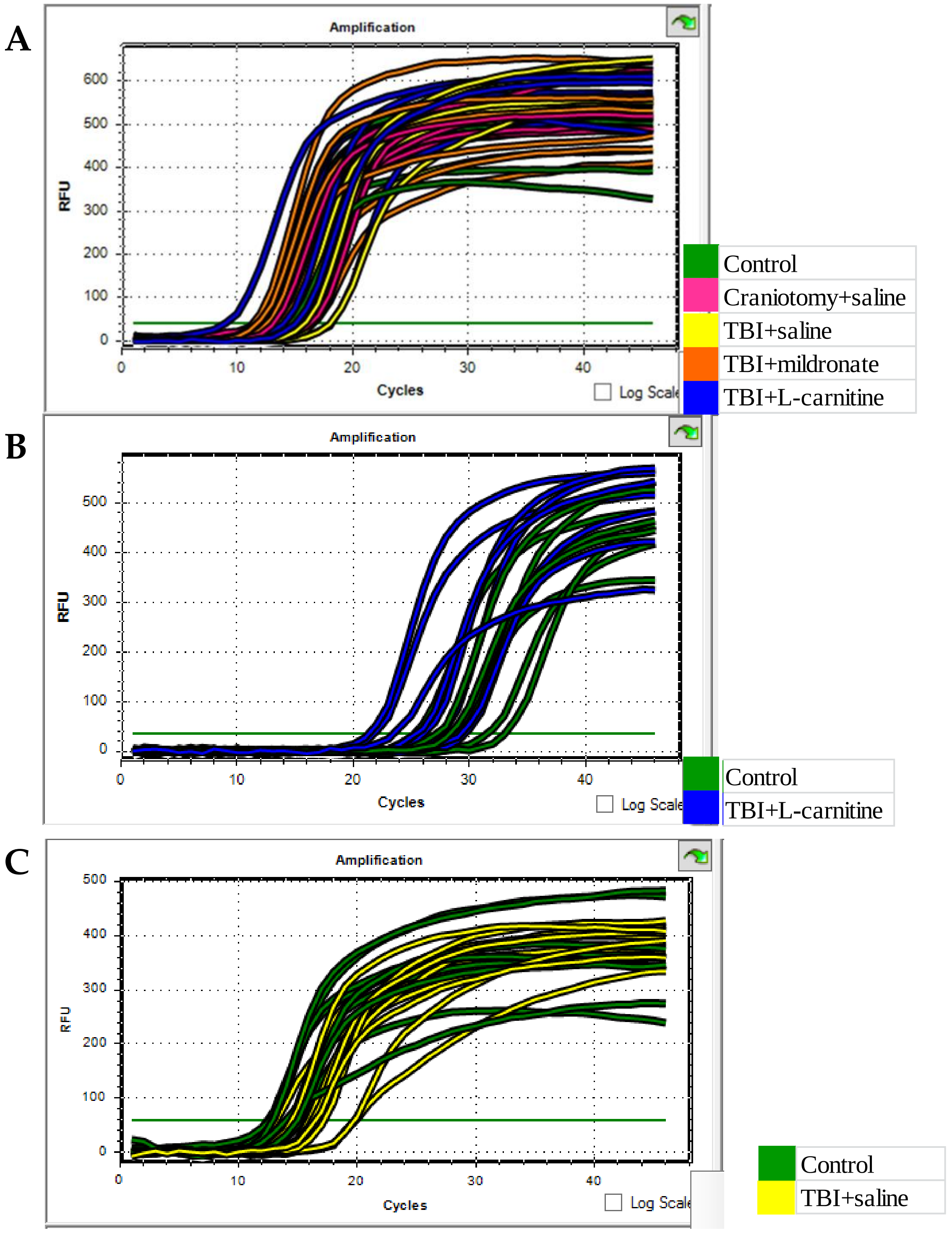

Figure S2. Representative examples of PCR product accumulation curves during estimation of bacterial levels in the gut microbiome. Universal (A), Tenericutes (B), Bacteroidetes (C) in relevant groups

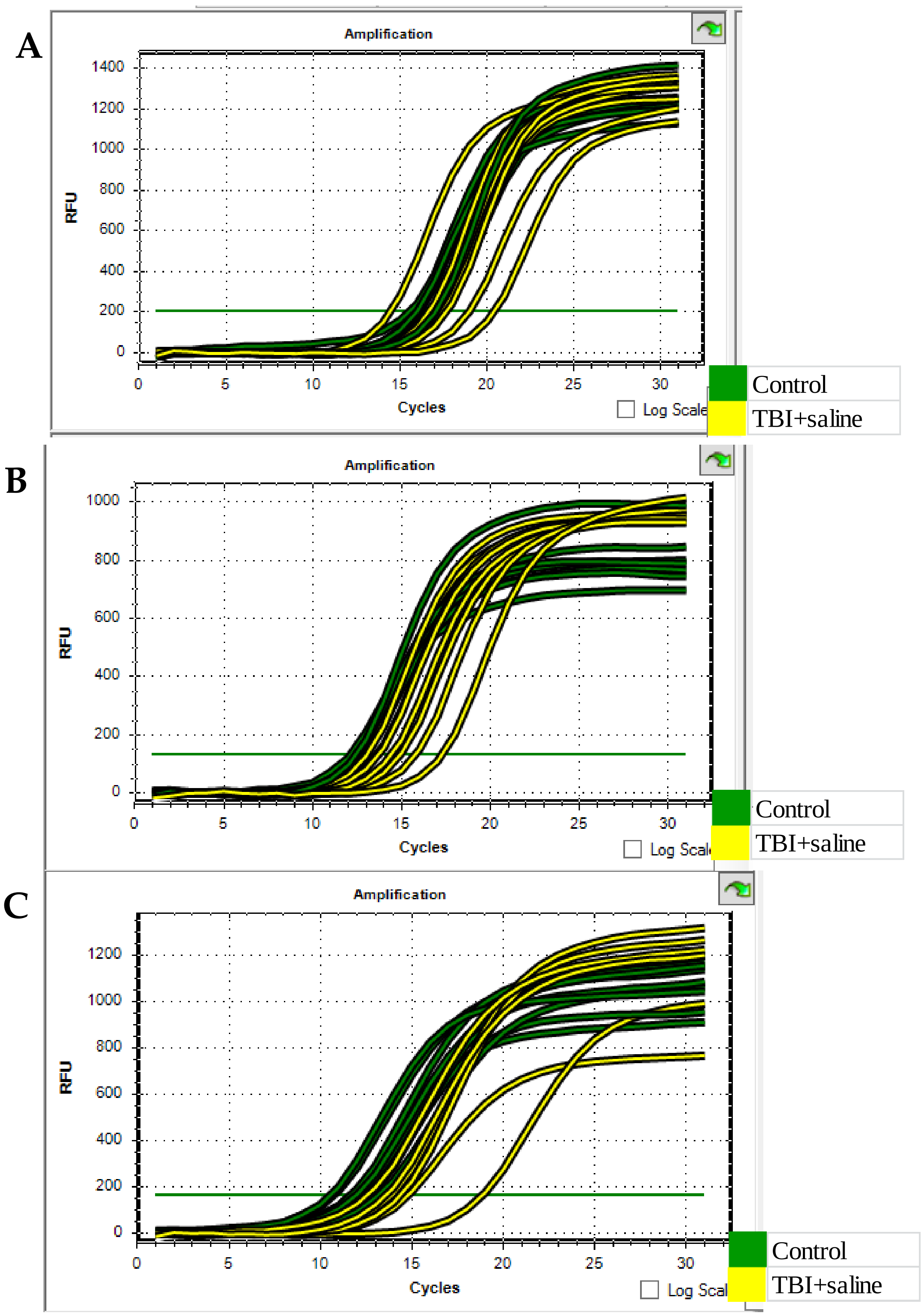

Figure S3. Representative examples of PCR product accumulation curves during mtDNA amplification in the Control and TBI + saline groups in the brain. Curves for *Gapdh* (nuclear reference) (A), short (mitochondrial) fragment (B), and long (mitochondrial) fragment (C).
